# The Ol' Switcheroo Shows How an RNA Enzyme Splices Itself

**DOI:** 10.1371/journal.pbio.0030310

**Published:** 2005-08-16

Ribonucleic acid (RNA) is a dogma breaker. The “central dogma” of cellular biochemistry mandates that deoxyribonucleic acid (DNA) stores information, and RNA copies this information and uses it to direct the assembly of amino acid building blocks into proteins, such as enzymes. Enzymes catalyze important chemical reactions in the cell, such as the breakdown of glucose or the synthesis of urea.

When biochemists discovered catalytic RNA, they had to ditch the dogma. Because of its structure, it turns out, RNA can act as an enzyme and catalyze reactions. While two strands of DNA tend to zip up into the famous double helix, RNA usually goes solo. The single RNA strand folds back on itself to create myriad tangled arrangements. Some of these arrangements create an active center, the place on the RNA where the enzymatic magic happens. The many RNA enzymes and protein enzymes that use metal atoms to do their job are called metalloenzymes. One example of an important structural motif in RNA metalloenzymes is the group I intron, which can snip itself out of an RNA segment. Understanding exactly how the RNA and the metals interact will help to provide precise answers about how the enzyme really works.

Through X-ray crystallography, researchers have revealed many structural features of group I introns. But X-ray crystallography creates images of the enzyme frozen in time; it does not catch an enzyme in action. In a new study, Joseph Piccirilli, Daniel Herschlag, and colleagues discovered that a particular oxygen atom on a particular nucleotide in a group I RNA must bind to a particular magnesium ion in order for the reaction under study to proceed normally. The oxygen atom is known as the *pro-S_P_* phosphoryl oxygen at nucleotide C262 in the intron from the unicellular Tetrahymena thermophila protozoan.

Since there's no way to watch the oxygen and metal hook up during the reaction, how do the researchers know they do? The researchers used the powerful techniques of metal ion rescue and atomic mutagenesis. Here's how it worked. They figured out how well the group I intron reaction works with a normal enzyme. Then, they replaced the oxygen in question with a sulfur atom. The reaction didn't work as well because, by the rules of chemistry, sulfur doesn't like to bind to magnesium. But sulfur does like manganese and cadmium ions. So they replaced the magnesium with one of these other metal ions and measured the reaction. The researchers saw that these other metal ions restored (or “rescued”) enzymatic activity. In short, the enzyme needs a bond where the oxygen and the magnesium are, but the bond doesn't have to be between oxygen and magnesium.

As complicated as that is, plucking out one atom and trading it for another is itself a tricky business. Because most enzymes are made of stubborn amino acids and not nucleotides, atomic mutagenesis can be difficult. And usually when researchers have tried atomic mutagenesis, they've mutated the substrate (the molecule that the reaction acts upon) instead of the enzyme (the molecule that acts). Here, Piccirilli, Herschlag, and colleagues directed the applications of atomic mutagenesis to the molecule that does the work. [Fig pbio-0030310-g001]

**Figure pbio-0030310-g001:**
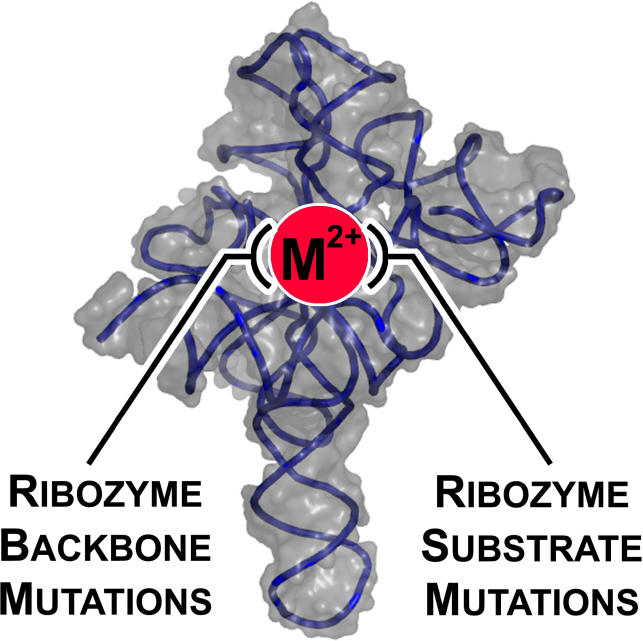
RNA enzymes called ribozymes require metal atoms to function. The site of metal-ribozyme interaction was studied by changing components of both the catalytic center of the ribozyme (the backbone) and its target substrate

To test that a specific oxygen in the intron binds to the magnesium ion, the researchers first had to compile a short list of potential atoms to which the magnesium might bind. By combining literature data from structural models and functional studies with a random sprinkling of sulfur atoms in the intron to find critical oxygen contacts, Piccirilli, Herschlag, and colleagues established a group of specific oxygen atoms to watch. They tried the metal rescue experiment with each of these oxygens, and the only enzyme rescued by the metal switch was the one in which they changed the C262 oxygen to a sulfur. Therefore, they concluded that this specific oxygen atom makes a critical contact with the magnesium ion. The strategy of atomic mutagenesis combined with metal ion rescue can be used to help understand the mechanism of other RNA and protein metalloenzymes.

